# Performance of the Interferon Gamma Release Assays in Tuberculosis Disease in Children Five Years Old or Less

**DOI:** 10.1371/journal.pone.0143820

**Published:** 2015-12-07

**Authors:** Lin Sun, Jian-ling Tian, Qing-qin Yin, Jing Xiao, Jie-qiong Li, Ya-jie Guo, Guo-shuang Feng, Xiao-xia Peng, Hui Qi, Fang Xu, Wei-wei Jiao, Chen Shen, A-dong Shen

**Affiliations:** 1 Key Laboratory of Major Diseases in Children and National Key Discipline of Pediatrics (Capital Medical University), Ministry of Education, National Clinical Research Center for Respiratory Diseases, Beijing Key Laboratory of Pediatric Respiratory Infection Diseases, Beijing Pediatric Research Institute, Beijing Children’s Hospital, Capital Medical University, Beijing, China; 2 Center for Clinical Epidemiology & Evidence-based Medicine, Beijing Children’s Hospital, Capital Medical University, Beijing, China; Hospital San Agustín. Aviles. Asturias. Spain, SPAIN

## Abstract

Interferon Gamma Release Assays (IGRAs) were developed for the indirect or immunologic diagnosis of tuberculosis infection; however, they have also been used to assist in difficult to diagnose cases of tuberculosis disease in adults, and to a lesser extent, in children, especially in those under 5 years old. We evaluated the utility of using an IGRA in pediatric tuberculosis in younger children in a hospital setting. The diagnostic accuracy of T-SPOT.*TB* and TST was assessed in 117 children with active tuberculosis and 413 children with respiratory tract infection. Sensitivity and specificity were calculated for the tests used individually and together. Concordance was also calculated. Sensitivity of T-SPOT.*TB* (82.9%) was higher than TST (78.6% using a 5mm cut-off), especially in children confirmed to have TB. T-SPOT.*TB* was more specific than TST using a 5mm cut-off (96.1% vs. 70.9%). Combining T-SPOT.*TB* and TST results improved the sensitivity to 96.6%. In conclusion, the results of the current study indicate that T-SPOT.*TB* has good sensitivity and specificity, supporting its use among patients of this age. A combination of IGRA and TST would be useful additions to assist in the diagnosis of childhood TB.

## Introduction

Timely and accurate diagnosis of tuberculosis (TB) disease in children must be given a high priority by medical practitioners for the following reasons: children carry 6% of the global burden of TB disease[1]; in 2002 around 9% of children in China were reported to be infected with *Mycobacterium tuberculosis* (MTB)[2]; children under 5 years old are more likely to develop the most severe forms of disseminated and meningeal TB [3], which is due to the immature immune system [4,5].

However, diagnosis of pediatric TB is challenging because symptoms are often non-specific, specimens may be difficult to obtain, and bacteriological confirmation is less frequent than in adults[6]. Furthermore, children younger than 5 are more likely to have severe extra-pulmonary TB while the most severe cases of TB are often seen in infants[7]. For these reasons, diagnosing TB in young infants warrants additional efforts.

Interferon gamma release assays (IGRAs), are promising alternatives to the tuberculin skin test (TST). However, few studies have investigated their use in young children and infants[8–10]. Consequently, guidelines from the American Academy of Pediatrics state that IGRAs are not recommended for routine use in children younger than five years of age due to a lack of published data[11].

IGRAs have been used mainly in the indirect or immunologic diagnosis of tuberculosis infection. They also can be used to assist in a diagnosis of tuberculosis disease in cases that are difficult to obtain a microbiological diagnosis or that need early diagnosis. Therefore, we have performed a study in a hospital setting to help provide this additional data by specifically answering the following questions: 1) what is the assistant diagnostic efficiency of TST and IGRA in children younger than 5 years old, and 2) can the accuracy of an IGRA be increased if it is used in conjunction with the TST?

## Methods

### Subjects

**Fig 1:**
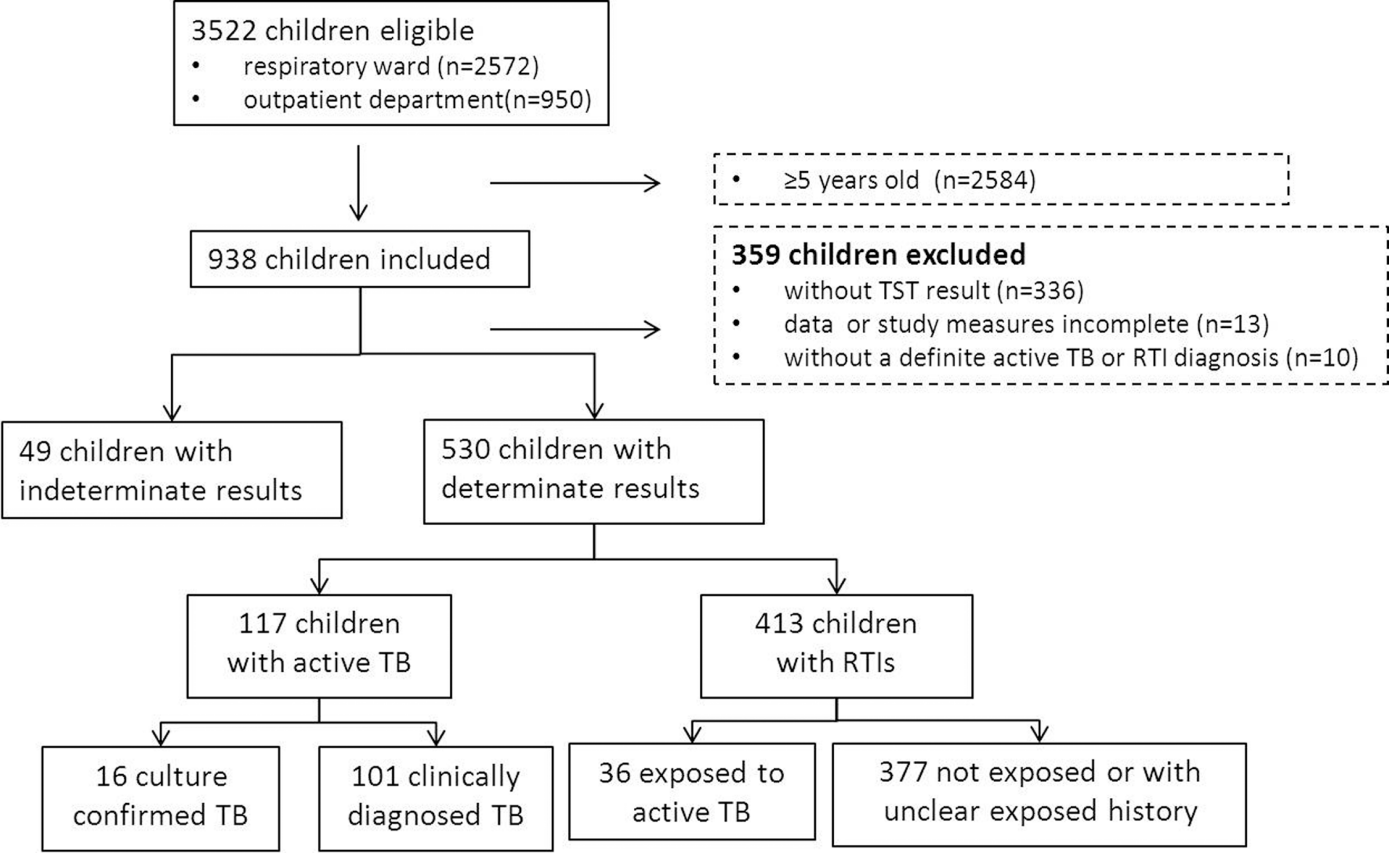
Flow diagram summarizing patient recruitment, exclusion criteria, and the patient groups.

The study was conducted among children evaluated for respiratory tract infection (RTI) and TB who were referred to the Beijing Children’s Hospital during the period from March 2011 through June 2014. Only children 5 years old and younger were included. Children with incomplete clinical data were excluded (Fig 1). The authors had access to identifying information during or after data collection. Clinical investigation had been conducted according to the principles expressed in the Declaration of Helsinki. This research has been approved by the Ethics Committee of Beijing Children’s Hospital. Written informed consent was obtained from the patients or the guardians of the patients that participated in this research.

### Case Group Definitions

Children were either classified as having tuberculosis or RTIs, the latter forming the control group in this study. Children with tuberculosis were divided into the subgroups listed below according to the presence of etiological evidence or disease severity.

TB was confirmed in children who had a positive culture result for MTB from sputum, bronchoalveolar lavage fluids, gastric washing, or cerebrospinal fluid. Clinical diagnosis of TB occurred in children who exhibited at least one symptom consistent with TB and who also had radiographic evidence consistent with TB.

Children diagnosed as having severe TB had two or more non-contiguous disease sites, miliary mottling, or involvement of meninges, pericardium, spinal, intestinal or splenic sites with or without lung involvement.

Children enrolled as controls were diagnosed as having RTIs other than tuberculosis if they presented with any of the following: 1) confirmed etiological evidence of infection other than MTB, e.g. viral diseases, mycoplasma, streptococcus pneumonia; 2) typical symptoms of RTIs which were not consistent with active TB; 3) symptoms that were alleviated without anti-tuberculosis treatment.

### Measurements

TST was performed using an intradermal injection of 5 IU purified protein derivative (PPD) from the Chengdu Institute of Biological Products, Chengdu, China. A trained pediatrician measured the transverse induration in millimeters 48–72 hours later.

The T-SPOT.*TB* test (Oxford Immunotec, Oxford, UK) was carried out according to the manufacturer’s recommendations, as previously described[12]. Briefly, 2–3 ml of blood was drawn from each subject, and peripheral blood mononuclear cells were separated and then were added to wells containing antigens or mitogen. The positive mitogen control was used to analyze general T-cell reactivity. The two testing wells were added with two MTB specific antigens, early-secreted antigenic target 6-kDa protein (ESAT-6) and culture filtrate protein 10 (CFP-10). No antigen was added to the negative control well as it was used to identify non-specific immune reactions. After incubating for 20 h, wells were washed and biotinylated anti-IFN-γmAb was added. After 1 h and additional washing, substrate was added and spots were enumerated by using an automated plate counter. The response was considered positive when either antigen well contained at least 6 more spots or twice the number of spots compared to the nil control well. Positive mitogen control with less than 20 spots was considered indeterminate.

### Statistical Analysis

Concordance between TST and IGRA in the different groups was assessed using percentage agreement and к coefficients. McNemar's test was used to evaluate the differences in sensitivity and specificity between the T-SPOT.*TB* and TST. The criterion for significance was set as *P*<0.05. Indeterminate results were excluded from the evaluation of accuracy.

## Results

### Study Participant Characteristics

Data from 579 children were included in the study. Demographic and socioeconomic characteristics of the 530 children with determinate results were documented (Table 1). Among them, 117 were classified as having active TB, and 413 children were diagnosed as having RTIs. There were 147 (27.7%) children less than 1 years of age. Twenty children (3.8%) were tested with HIV in hospital and received a negative result, and the other 510(96.2%) children were denied to be HIV infected by their patients. Children with HIV infection were excluded.

**Table 1:** Main clinical characteristics of the study population.

Characteristic	Total (N = 530), n (%)	Children with Active TB*(N = 117), n (%)	Children with RTIs(N = 413), n (%)	*P* value
**Age**				0.077
** 0–12 months**	147 (27.7)	41 (35.0)	106 (25.7)	
** 1–2 years**	190 (35.8)	42 (35.9)	148 (35.8)	
** 3–5 years**	193 (36.4)	34 (29.1)	159 (38.5)	
**Gender**				0.032
** Male**	326 (61.5)	62 (53.0)	264 (63.9)	
** Female**	204 (38.5)	55 (47.0)	149 (36.1)	
**BCG vaccination**				0.001
**Yes**	486 (91.7)	94 (80.3)	392 (94.9)	
** No**	35 (6.6)	22 (18.8)	13 (3.1)	
**Unclear**	9 (1.7)	1 (0.9)	8 (1.9)	
**History of exposure**				0.001
** Yes**	79 (14.9)	43 (36.8)	36 (8.7)	
** No**	440 (83.0)	73 (62.4)	367 (88.9)	
**Unclear**	11 (2.1)	1 (0.9)	10 (2.4)	
**Location**				0.037
** Rural**	296 (55.8)	79 (67.6)	217 (52.5)	
** City**	129 (24.3)	25 (21.4)	104 (25.2)	
** Town**	105 (19.8)	13 (11.1)	92 (22.3)	

* Confirmed TB and clinically diagnosed TB.

### Evaluation of Diagnostic Accuracy

Sensitivity data is shown in Table 2. Sensitivity of the T-SPOT.*TB* test was higher than the TST even when using a 5mm cut-off (100% vs 81.3% in the confirmed group and 80.2% vs 78.2% in the clinically diagnosed group). Sensitivity of both tests increased with the age of the children. Sensitivity of both tests was higher in children with non-severe TB compared to severe TB (89.8% vs 75.9% for the T-SPOT.*TB* test and 88.1% vs 69.0% for the TST using a 5mm cut-off).

**Table 2:** Sensitivity of T-SPOT.TB and TST tests in children with confirmed and clinically diagnosed tuberculosis.

	No. of subjects	T-SPOT.*TB*	TST					
		Sens,%	≥5mm		≥10mm		≥15mm	
			Sens,%	P* value	Sens,%	P* value	Sens,%	P* value
**Total**	117	82.9	78.6	0.407	67.5	0.006	29.9	<0.001
**Age**								
** 0–12 months**	41	78.0	65.9	0.219	46.3	0.003	19.5	<0.001
** 1–2 years**	42	81.0	83.3	0.776	73.8	0.434	28.6	<0.001
** 3–5 years**	34	91.2	88.2	0.690	85.3	0.452	44.1	<0.001
**Diagnostic standard**								
** Confirmed TB**	16	100.0	81.3	0.226	75.0	0.101	37.5	<0.001
** Clinically diagnosed TB**	101	80.2	78.2	0.729	66.3	0.026	28.7	<0.001
**Severity**								
** Non severe TB**	59	89.8	88.1	0.769	69.5	0.006	28.8	<0.001
** Severe TB**	58	75.9	69.0	0.406	65.5	0.221	31.0	<0.001

Table 3 indicates the specificity of T-SPOT.*TB* test and TST for active TB disease using the control group of children with RTIs. T-SPOT.*TB* was more specific than TST in each of the age subgroups. Both tests had fewer false positive results in children less than one year old.

**Table 3:** Specificity of the T-SPOT.TB and TST for active TB disease.

**Age**	No. of subjects	T-SPOT.*TB*	TST					
		Spec,%	≥5mm		≥10mm		≥15mm	
			Spec,%	P* value	Spec,%	P* value	Spec,%	P* value
**Total**	413	96.1	70.9	<0.001	75.3	<0.001	91.5	0.006
**0–12 months**	106	99.1	86.8	<0.001	86.8	<0.001	97.2	0.313
**1–2 years**	148	94.6	69.6	<0.001	73.0	<0.001	87.8	0.112
**3–5 years**	159	95.6	61.6	<0.001	69.8	<0.001	91.2	0.114

**Fig 2:**
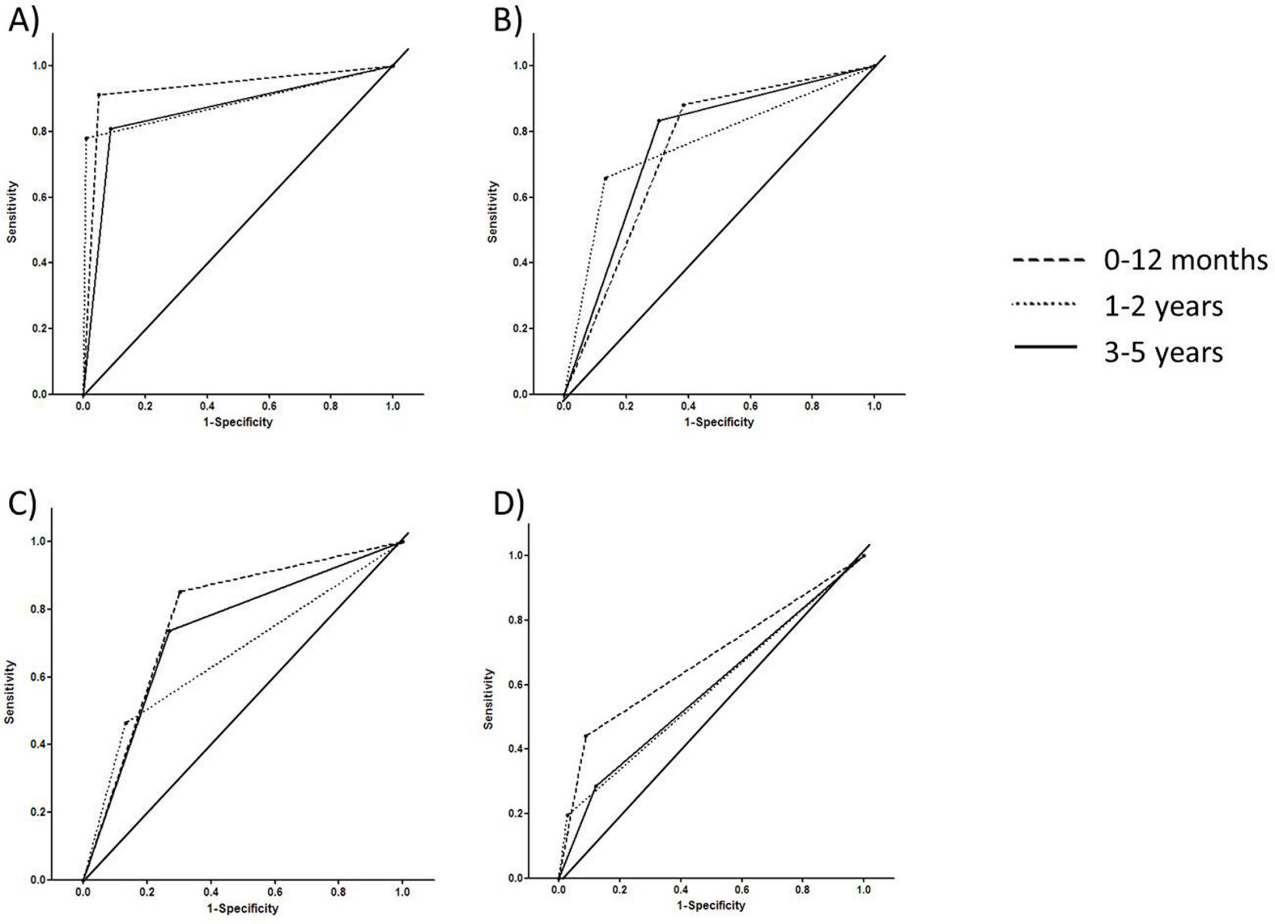
Area under the curve (AUC) for T-SPOT.*TB* and TST. This figure shows receiver-operating-characteristic (ROC) curves for the sensitivity and specificity of T-SPOT.TB (A) and the TST tests using 5mm, 10mm and 15mm cut-offs (B-D).

The diagnostic accuracy of the tests in the three age-groups was then compared (Fig 2). There was no significant difference in the area under the curve (AUC) between the three age groups for T-SPOT.*TB*. The T-SPOT.*TB* test had a higher AUC than TST in each of the age groups, indicating that it had better diagnostic accuracy.

### Concordance between T-SPOT.*TB* and TST tests

A 10mm cut-off was used in the concordance analysis. As shown in Table 4, total concordant was poor both in the active TB and in the RTI groups (*Kappa* <0.4).

In children with active TB, the concordance of T-SPOT.*TB* and TST increased with age, with the most significant cause of discordant results being in children with a positive T-SPOT.*TB* result but a negative TST result. In children with RTIs concordance decreased with increasing age with the most significant cause of discordant results being in children with a negative T-SPOT.*TB* result but a positive TST result.

**Table 4:** Concordance between T-SPOT.TB and TST (10mm cut-off) for children of different ages.

Age (years)	Concordant results, n (%)			Discordant results, n (%)			Kappa
	TSPOT+TST+	TSPOT-TST-	Total, %	TSPOT+TST-	TSPOT-TST+	Total, %	
**Children with active TB**							
** 0–12 months**	16 (39.0)	6 (14.7)	22 (53.7)	16 (39.0)	3 (7.3)	19 (46.3)	0.110
** 1–2 years**	24 (57.1)	1 (2.4)	25 (59.5)	10 (23.8)	7 (16.7)	17 (40.5)	-0.148
** 3–5 years**	26 (76.5)	0 (0)	26 (76.5)	5 (14.7)	3 (8.8)	8 (23.5)	-0.124
** Total**	66 (56.4)	7 (6.0)	73 (62.4)	31 (26.5)	13 (11.1)	44 (37.6)	0.022
**Children with RTIs**							
** 0–12 months**	1 (0.9)	92 (86.8)	93 (87.7)	0 (0)	13 (12.3)	13 (12.3)	0.118
** 1–2 years**	10 (6.8)	105 (70.9)	115 (77.7)	3 (2.0)	31 (20.3)	33 (22.3)	0.274
** 3–5 years**	7 (4.4)	110 (69.2)	117 (73.6)	1 (0.6)	41 (25.8)	42 (26.4)	0.179
** Total**	18 (4.4)	307 (74.3)	325 (78.7)	4 (1.0)	84 (20.3)	88 (21.3)	0.692

The discordant T-SPOT.*TB*/TST results in the two subgroups were then examined. In the active TB group, 25 children had a TST induration diameter from 0mm to 5mm, 21/25 (84%) of them were T-SPOT.*TB* positive. In RTIs group, 22 children had a positive T-SPOT.*TB* result, 18/22 (81.8%) had a TST result greater than 10mm, 40.9% (9/22) of these had a TST result between 10mm and 14mm.

**Fig 3:**
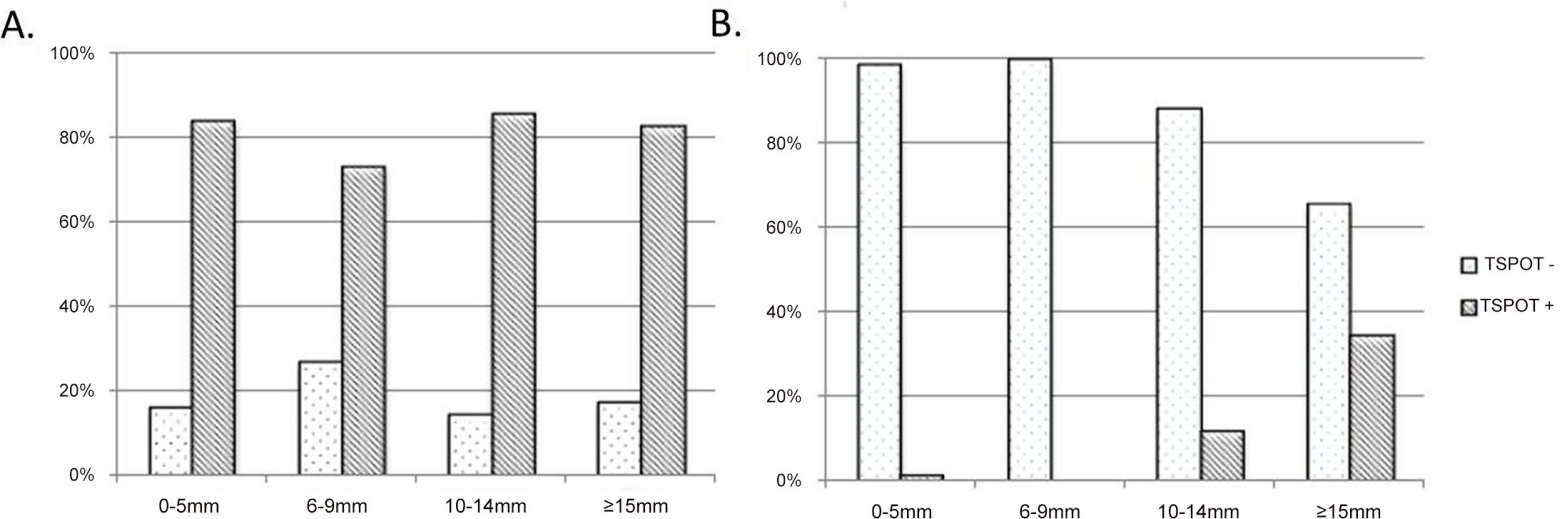
Distribution of positive T-SPOT.*TB* results in active TB (A) and RTIs (B) children with different TST induration diameter.

We then compared the distribution of positive T-SPOT.*TB* results in children with different TST indurations (Fig 3). In active TB children, T-SPOT.*TB* positivity rate is similar in the four subgroups, while in the RTI group, positive T-SPOT.*TB* results were mostly observed in children with lager TST induration diameter (11.9% in 10–14mm subgroup and 34.3% in ≥ 15mm subgroup).

### Combining tests to optimize results

To maximize the sensitivity of the test regimen, the effect of combining the T-SPOT.*TB* and TST results was examined (Table 5). The effect of combining the results of the T-SPOT.*TB* test with the 3 TST cut-offs is then compared. The highest sensitivity (95.7%) was achieved when the T-SPOT.*TB* test was combined with the TST using a 5mm cut-off. However, this combination of tests resulted in a low specificity (70.2%).

**Table 5:** Sensitivity of T-SPOT.*TB* and TST tests (singly and combined) in children with confirmed and clinically diagnosed tuberculosis using different cut-offs for TST.

	Sensitivity n (%)	Specificity n (%)
**T-SPOT.*TB***	97/117 (82.9)	397/413 (96.1)
**TST 5mm**	92/117 (78.6)	293/413 (70.9)
**TST 10mm**	79/117 (67.5)	311/413 (75.3)
**TST 15mm**	35/117 (29.9)	378/413 (91.5)
**T-SPOT.*TB* or TST 5mm** *	112/117 (95.7)	290/413 (70.2)
**T-SPOT.*TB* or TST 10mm** *	110/117 (94.0)	304/413 (73.6)
**T-SPOT.*TB* or TST 15mm** *	101/117 (86.3)	354/413 (85.7)

*Either or both tests could be positive for a diagnosis of TB. Both tests had to be negative to exclude TB.

### Indeterminate T-SPOT.TB Results

The overall proportion of indeterminate results was high (8.5%, 49/579). 38 (77.6%) were due to low positive control, while 11(22.4%) were due to high nil control. Indeterminate rates were higher (10.2%) in children younger than 1 year old. Of the 49 children with indeterminate results, 4 (8.1%) were diagnosed as having severe TB, and 12 (24.5%) were diagnosed as having severe mycoplasma pneumonia, refractory mycoplasma, or necrotizing pneumonia.

## Discussion

The accuracy and reliability of IGRAs among children five years old and younger in hospital settings is not yet well defined. The most important feature of our study is that all of the children enrolled were younger than 5 years of age, and so the findings of this study have implications for the use of T-SPOT.*TB* in supporting the diagnosis of tuberculosis in young children.

Our study showed that T-SPOT.*TB* is sensitive in older children (aged 3–5 years old) and in all children with culture confirmed TB. There are some inconsistencies between different studies concerning the diagnostic accuracy of T-SPOT.*TB* even in children confirmed to have TB. Schopfer[13] concluded that the IGRA test cannot be used as a ‘rule-in’ test because of its low sensitivity in culture confirmed children. A recently published meta-analysis reported that T-SPOT.*TB* sensitivity reached 80% in microbiologically confirmed cases[14]. Similarly, the comparative performance of the test in the very young is controversial. One study which enrolled children with TB aged under 5 years old in a community setting, reported that T-SPOT.*TB* had a lower sensitivity than in older children[15], while Critselis reported that the accuracy of IGRA is not affected by age[9].

When a TST cut-off of 5mm is used, its sensitivity is close to that of the TSPOT.*TB* test but the specificity of the TST is very poor when this cut-off is used. Almost all recent studies provide support for the specificity of IGRAs in screening for pediatric TB. Our data confirmed the IGRAs value to discriminate between TB and a false positive TST reaction. BCG vaccination is almost universal in China. Additionally, there has been a rapid increase in NTM infections in recent years. In the absence of definitive evidence to confirm TB or cross reactivity with BCG or NTM, a clinician would assume that a weaker TST (<10mm) was more likely to be associated with cross reactivity to BCG, while an induration larger than 15mm would suggest tuberculosis. However, in our study, in children from the control group who had a TST induration larger than 15mm, most were negative by the T-SPOT.*TB* test suggesting that even large TST indurations may be caused by cross reactions. This conclusion is supported by a study which reported that in approximately 50% of children with NTM diseases the TST induration exceeded 15mm [16]. Therefore, using only the TST may result in over diagnosis and overtreatment of children so using an IGRA can be helpful to eliminate false positive TST results in children from countries with a high rate of BCG vaccination and NTM infections.

In our study, the concordance between the T-SPOT.*TB* test and the TST was low both in children with active TB and those with RTIs. In children with active disease the concordance between the tests increased with age. In the very young children (0–12 months) the discordance was more likely to be TST-ve/T-SPOT.*TB*+ve indicating the higher sensitivity of the T-SPOT.*TB* test in the very young. Conversely, concordance decreased with age in children with RTIs. Two factors are known to produce false positive TST results; BCG vaccination and NTM infections. Firstly, as BCG vaccination is carried out in the new-born in China, the effect of BCG on the TST would be expected to diminish with age so there would be more discordance between the tests in the younger children. Secondly, as children grow older they are increasingly likely to have been infected with NTMs, thereby increasing false positive TST results. Our results suggest that the NTM effect outweighs the BCG effect in this population.

To increase sensitivity the results of both tests could be combined. A positive result from either test would indicate TB infection. However, the use of this combined testing methodology would be expected to reduce specificity. This was seen in our data. When using a 5mm TST cut-off the sensitivity of the combined testing regimen (95.7%) was greater than the sensitivity of the individual tests (82.9% for T-SPOT.*TB* and 78.6% for the TST). However, the specificity when combining the results of the tests (70.2%) was lower than that for the individual tests (96.1% for T-SPOT.*TB* and 70.9% for the TST).

Some studies have reported high numbers of indeterminate IGRA results in children, especially in younger children. This has led to a number of national guidelines suggesting that IGRAs should be used with caution in children younger than five years old. In the present study, 8.5% of results were indeterminate. In previous pediatric studies 0–35% of IGRA results were indeterminate[8,10,17]. Previously reported risk factors associated with indeterminate results include young age, immune deficiency[18], poor diet and helminth infections[19]. No conclusion has been reached concerning the effect of young age on indeterminate IGRA results[20,21]. Other factors that affect the rate of indeterminate results include which particular IGRA is being used, the subjects being tested, various factors that degrade samples between blood collection and laboratory processing and the quality of the laboratory running the test. We believe the indeterminate results in this study may have been caused by the very young age of many of the subjects and the poor health of some of the subjects due to severe tuberculosis or other pulmonary diseases.

There were a number of limitations to this study. Firstly, only 16 of the 117 children considered to have TB were confirmed using culture. The remainders were clinically diagnosed. This is a common issue with all pediatric TB studies due to the difficulty in obtaining a positive culture result in young children. It is therefore possible that some of the subjects classified as having TB may have had other illness with similar symptoms. This would explain why both tests had higher sensitivity in the culture confirmed sub-group. Secondly, both the tests used in the study will identify both active disease and LTBI. RTI children with positive T-SPOT.*TB* results were mostly observed to have lager TST induration diameter, suggesting that the concordant positive results were caused by children who were latently infected with TB. It is therefore likely that some of the subjects from the RTI group who were positive by one or both of the tests had LTBI. Finally, it was not possible to identify if any of the children had received one or more prior TSTs. Since it is possible that prior TSTs may boost the TST itself and, to a lesser extent, an IGRA this may have induced some false positive results. However, the children tested were representative of a “real life” situation so such boosting, if it occurred, would also be present in clinical situations so these results would still be representative.

## Conclusions

The data from this study suggests that the T-SPOT.*TB* test is well-suited to assist in the diagnosis of tuberculosis in children up to five years old. We have shown that a combination of IGRA and TST provides greater sensitivity at the expense of specificity. In young children identification of active disease is critical and difficult so this would be an acceptable compromise. Therefore the use of the T-SPOT.*TB* test would be a useful addition in the diagnosis of childhood TB. Since resources are often limited, further studies are needed to determine the most cost effective combination of these tests when diagnosing children taking into account their age, clinical symptoms and immune status.
